# *DICER1* somatic mutations strongly impair miRNA processing even in benign thyroid lesions

**DOI:** 10.18632/oncotarget.26639

**Published:** 2019-03-05

**Authors:** Anello Marcello Poma, Vincenzo Condello, Maria Denaro, Liborio Torregrossa, Rossella Elisei, Paolo Vitti, Fulvio Basolo

**Affiliations:** ^1^ Department of Surgical, Medical, Molecular Pathology and Critical Area, University of Pisa, Pisa, Italy; ^2^ Section of Pathology, University Hospital of Pisa, Pisa, Italy; ^3^ Department of Clinical and Experimental Medicine, Unit of Endocrinology, University of Pisa, Pisa, Italy

**Keywords:** thyroid cancer, *DICER1*, miRNA, mutation, follicular adenoma

## Abstract

The alteration of miRNA processing is a driver event in several tumors including thyroid cancer. In particular, somatic *DICER1* mutations, reported in follicular-patterned lesions, are shared by benign as well as malignant tumors. In the present study, we investigated the effects of alterations in the miRNA processing genes on the miRNA profile.

The study included 19 follicular adenomas (FAs) and 22 follicular variant of papillary thyroid carcinomas (FVPTCs). The mutational status in the hot spot regions of *DICER1*, *DROSHA*, *TARBP2*, *DGCR8* and the most commonly affected genes in thyroid tumors was investigated on both tumor and paired normal tissues. The miRNA profile and the mRNA expression levels of *DICER1*, *DROSHA*, *TARBP2*, *DGCR8* and *XPO5* were also evaluated.

Two *DICER1* RNase IIIb domain mutations were found in FAs. These lesions presented a considerable loss of 5p miRNAs. Fifteen miRNAs were specifically deregulated in *DICER1*-mutant lesions compared to FAs and FVPTCs. These miRNAs regulate crucial pathways in cancer such as Hippo, p53 and TGF-beta signalling.

*DICER1* somatic mutations in the RNase IIIb domain are not specific for malignancy, but the miRNA imbalance that they cause is remarkable, especially with regard to the loss of 5p miRNAs. *DICER1*-mutant lesions have a characteristic miRNA deregulation, which is different from that of FVPTCs; nevertheless, this impairment is consistent with malignant transformation. Further studies providing the real risk of malignancy associated with *DICER1* mutations and the evolution of *DICER1*-mutant lesions are needed to make them useful in the clinical practice.

## INTRODUCTION

The biogenesis of microRNAs (miRNAs) occurs in the nucleus and in the cytoplasm. In the nucleus, the microprocessor complex, composed by the ribonuclease DROSHA and by the RNA binding protein DGCR8 (DGCR8, microprocessor complex subunit), cleaves the primary transcripts of miRNAs (pri-miRNAs) in pre-miRNAs of about 70 nucleotides. Pre-miRNAs exit the nucleus by exportin 5 (XPO5), and in the cytoplasm they undergo further processing by the ribonuclease DICER1 and the RNA binding protein TARBP2 (TARBP2, RISC loading complex RNA binding subunit) [1]. This step produces mature miRNAs that will be assembled in the RNA-induced silencing complex in order to silence the expression of specific targets by translational repression, mRNA deadenylation and decay [2].

The alteration of this machinery has been reported in several human cancers; however, the extent of this dysregulation is controversial. In fact, the effect of the up- or down-regulation of the miRNA processing genes seems to be tissue-specific [1, 3]. For instance, in some tumors like acute myeloid leukaemia, prostate and oral cancers, DICER1 is overexpressed both at the mRNA and the protein level. On the contrary, in other tumors such as lung, skin and breast cancers, low levels of DICER1 mRNA or protein are associated with malignant lesions and poor prognosis [1].

Alongside expression alterations, mutations in the genes involved in the processing of miRNAs have been reported both at the somatic and germline levels [1, 3–5]. In particular, germline mutations in *DICER1* predispose individuals to multiple tumors, benign and malignant alike, including pleuropulmonary blastoma, cystic nefroma, embryonal rhabdomyosarcoma and multinodular goiter [1, 6]. As regards thyroid disorders, germline mutations in *DICER1* are associated with a higher risk to develop not only benign conditions but also well-differentiated carcinomas [7]. Furthermore, somatic mutations, especially in the RNase IIIb domain, are potential driver events in well-differentiated thyroid cancer [8–10], probably leading to an imbalance in miRNA processing and in particular to a consistent loss of mature 5p miRNAs [11].

In this complex and variable scenario, we investigated the presence of mutations in the hot spot regions of genes involved in miRNA processing in follicular-patterned thyroid tumors. In detail, we focused on crucial regions considering both the functional domains and the presence of somatic mutations described in the literature. We also tested the presence of mutations in the most commonly affected genes in thyroid cancer. Finally, we evaluated the mRNA expression levels of *DICER1*, *DROSHA*, *TARBP2*, *DGCR8* and *XPO5*, and the miRNA expression profile.

## RESULTS

### Clinico-pathological features

Among the 41 follicular patterned thyroid lesions, there were 19 follicular adenomas (FAs) and 22 follicular variant of papillary thyroid carcinomas (FVPTCs). None of the cases presented oncocytic aspects. Among the 22 FVPTCs, nine were encapsulated non-invasive, eight encapsulated invasive, and five infiltrative. None of the encapsulated non-invasive lesions met the diagnostic criteria of non-invasive follicular thyroid neoplasms with papillary-like nuclear features [12, 13]. Overall, there were nine males and 32 females, the mean age was 44.4 ± 15.5 years and the mean size was 36 ± 16 mm. There were no differences between FAs and FVPTCs in terms of gender, age and size.

### Mutational status and mutation prediction

Overall, 14 somatic and four germline mutations were found in heterozygosis. Briefly, eight out of 14 somatic mutations were *NRAS*, four *HRAS* and two *DICER1*. No *PAX8-PPARG* rearrangements were detected, and no mutations were found in *BRAF*, *KRAS*, *DGCR8* and *TARBP2*. Both *DICER1* mutations were detected in FAs. All germline mutations were synonymous *DROSHA* variants, two were p.S981S and two were p.Y1199Y. Two *DROSHA* germline mutant carriers also harbored *RAS* mutations. Three out of 14 somatic mutations were found in FAs, whereas 11 were detected in FVPTCs. Germline mutations were equally split between FAs and FVPTCs. Details are shown in Table 1. Germline *DROSHA* mutations were already reported as single nucleotide variants, rs17485810 (p.S981S) and rs61748189 (p.Y1199Y). However, the minor allele frequencies on Exome Aggregation Consortium (ExAC) [14] and 1000 Genomes [15] were 0.0032 and 0.0028 for rs17485810, and 0.0080 and 0.0038 for rs61748189, respectively. Both variants were predicted to be disease causing by Mutation Taster (without amino acid exchange model, prob=1); in detail, p.S981S affects a splicing site, whereas p.Y1199Y involves a CG-rich region, potentially affecting Histone 3 Lysine 36 Tri-Methylation. Patients carrying *DROSHA* germline mutations were younger than the others, but the p value was not significant (p=0.0633).

**Table 1:** Mutational status

Histology	*NRAS*	*HRAS*	*DICER1*	*DROSHA*
FAs (n=19)	1 p.Q61R (c.182 A>G)	-	1 p.D1810V (c.5429 A>T)1 p.E1813K (c.5437 G>A)	1 p.S981S (c.2943 C>T) 1 p.Y1199Y (c.3597 C>T)
FVPTCs (n=22)	7 p.Q61R (c.182 A>G)	4 p.Q61R (c.182 A>G)		1 p.S981S (c.2943 C>T) 1 p.Y1199Y (c.3597 C>T)

FAs follicular adenomas; FVPTCs follicular variant of papillary thyroid carcinomas.

### miRNA expression analyses

**Figure 1:**
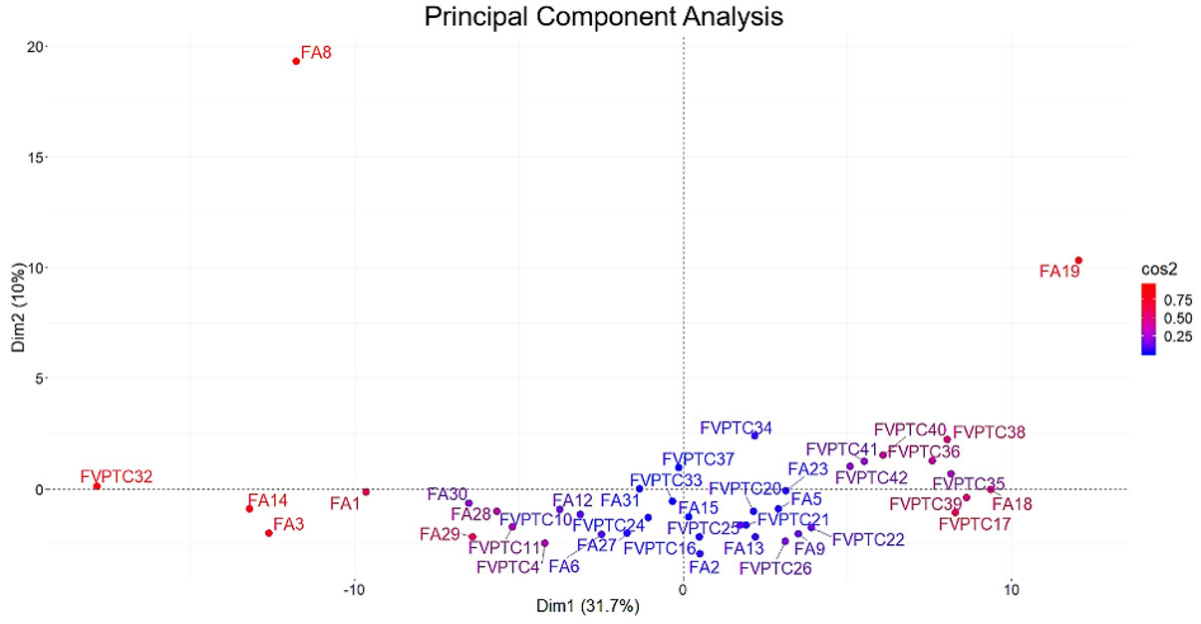
Principal component analysis All the 143 miRNAs considered were used in the analysis. Principal components 1 and 2 accounted for 41.7% of variability and were used for plotting. *DICER1* mutated cases (FA8 and FA19) mostly contributed to the variance as highlighted by squared cosine (cos2).

**Figure 2:**
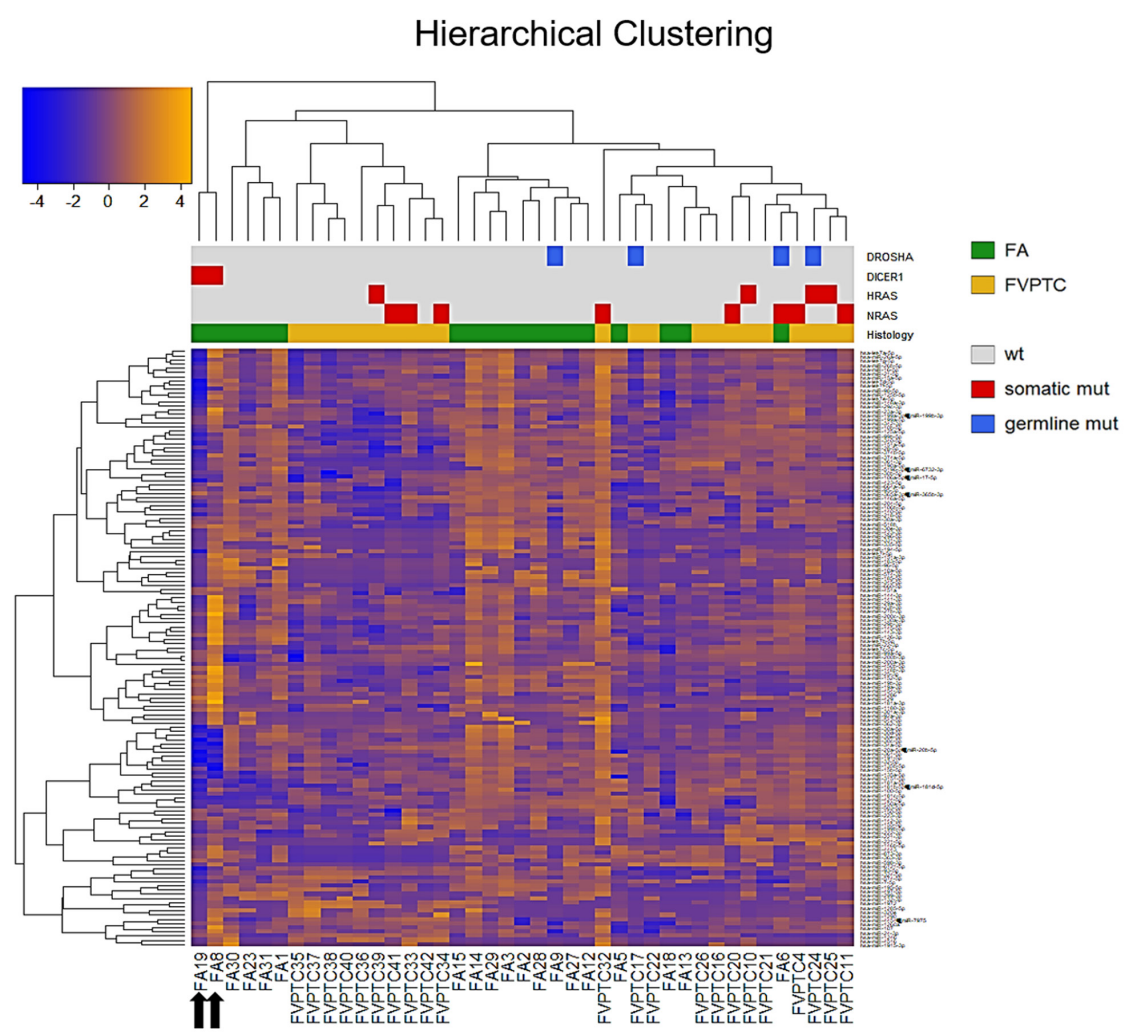
Hierarchical clustering All the 143 miRNAs (rows) and samples (columns) were independently clustered using Pearson’s correlation. Sidebars show the histological class and the mutational status. Black arrows emphasize the two *DICER1* mutant FAs. FA follicular adenoma, FVPTC follicular variant of papillary thyroid carcinoma.

**Figure 3:**
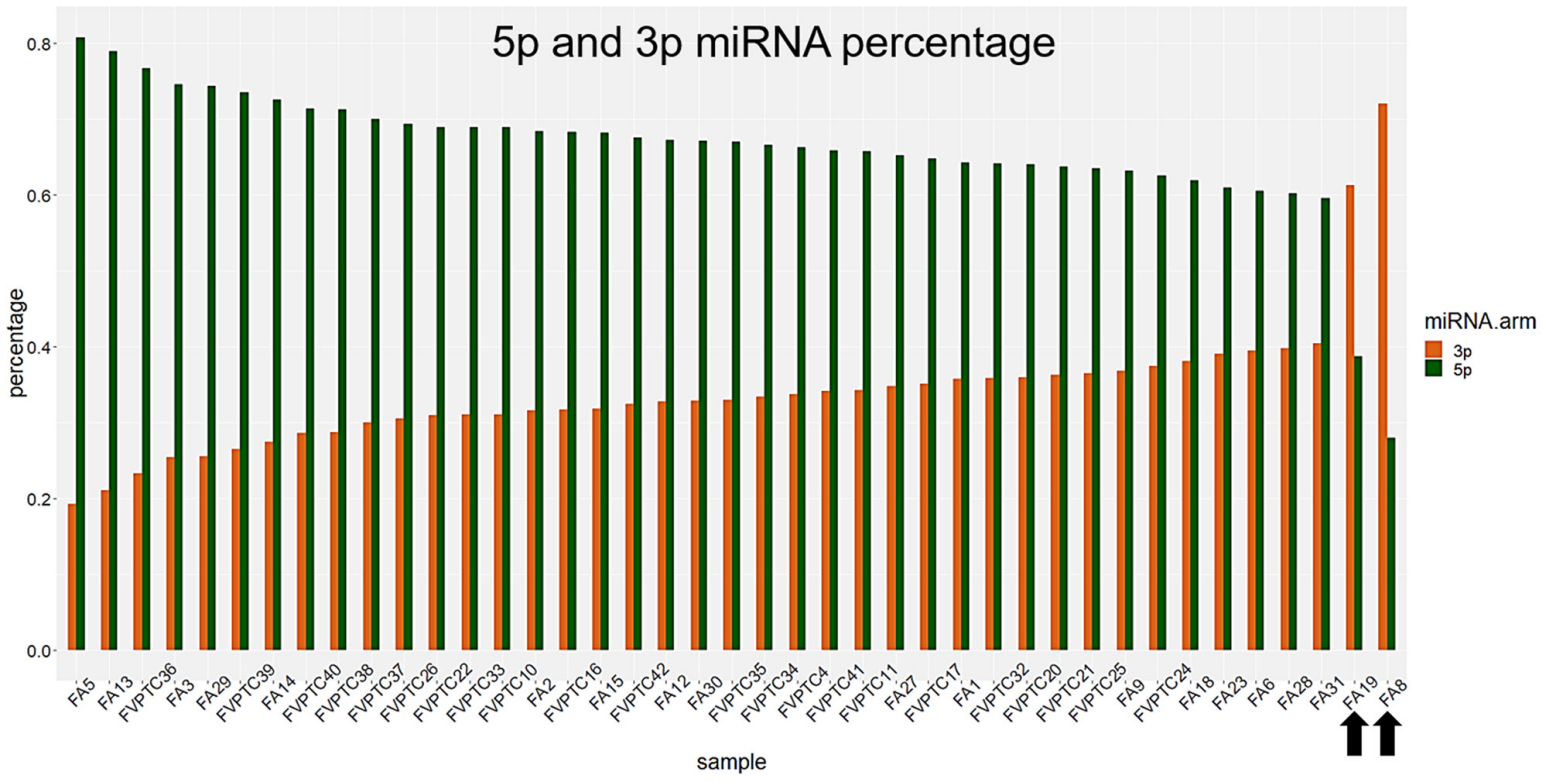
3p and 5p percentages Percentages were defined as the amount of 3p and 5p miRNAs out of the total. *DICER1* mutated cases, highlighted by black arrows, had a remarkable reduction of 5p miRNAs.

A total of 143 miRNAs were considered after normalization. The principal component analysis (PCA) produced 41 principal components; the first two accounted for 41.7% of variance and were used for plotting. As illustrated in Figure 1, FA8 and FA19, both harboring *DICER1* mutations, mostly contributed to the variance. Hierarchical clustering showed that the miRNA profiles of the two *DICER1* mutated cases were highly correlated (Figure 2). 5p and 3p percentages were defined as the proportion of cumulative 5p and 3p miRNA normalized counts on the total of normalized miRNA counts per sample, respectively. All samples had a predominance of 5p miRNAs, except for the two *DICER1-*mutant FAs (Figure 3).

**Figure 4:**
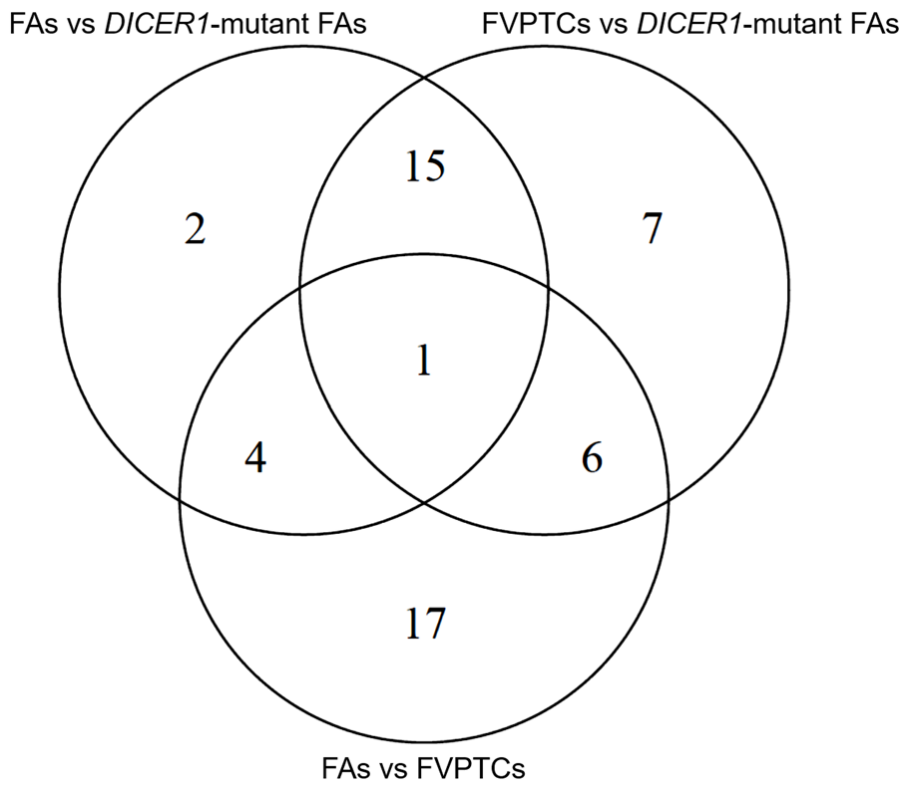
Venn diagram of differentially expressed miRNAs

**Figure 5:**
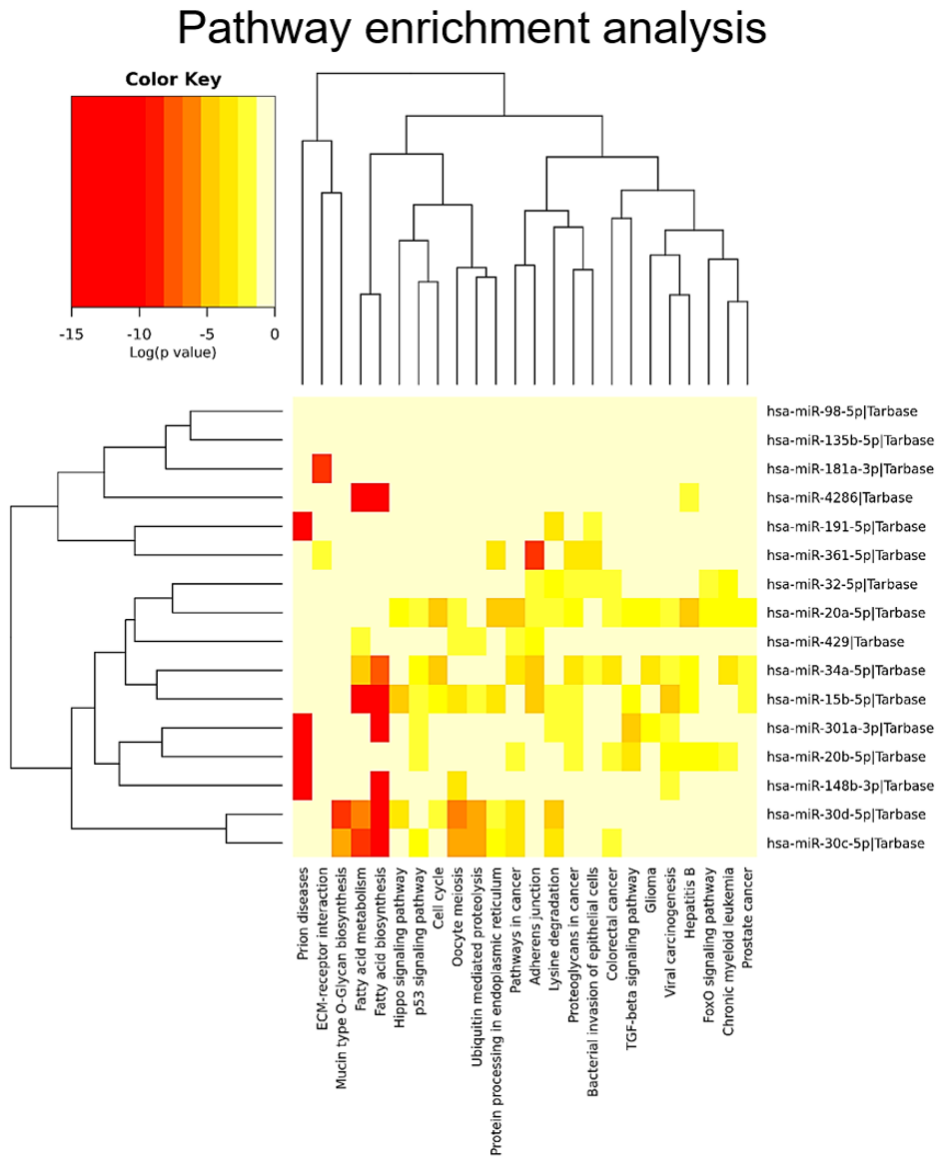
Pathway enrichment analysis The 15 *DICER1*-specific miRNAs were used in the analysis to predict the pathways that could be affected.

Twenty-two, 29 and 28 miRNAs were differentially expressed by contrasting FAs versus *DICER1*-mutant FAs, FVPTCs versus *DICER1*-mutant FAs and FAs versus FVTPCs, respectively. Some of these miRNAs were differently expressed in more than one comparison, but 17 were specifically deregulated in FVPTCs compared to FAs. Finally, 15 miRNAs were deregulated in *DICER1*-mutant FAs versus both *DICER1*-negative FVPTCs and FAs (Figure 4 and Table 2). These 15 miRNAs were then tested for pathway enrichment analysis to highlight pathways deregulated in *DICER1*-mutant lesions. Twenty-four pathways were enriched, and fatty acids synthesis and metabolism were among those with the highest confidence. In addition, crucial pathways in cancer such as Hippo, p53 and TGF-beta signalling were also enriched (Figure 5).

**Table 2:** miRNA differential expression analysis

	FAs vs *DICER1*-mutant FAs		FVPTCs vs *DICER1*-mutant FAs		FAs vs FVPTCs	
miRNA	log FC	FDR	log FC	FDR	log FC	FDR
hsa-miR-106a-5p + hsa-miR-17-5p	-3.32	0.0009				
hsa-miR-106b-5p	-4.47	0.0079			-2.07	0.0281
hsa-miR-126-3p			2.57	0.0100	-0.81	0.0343
hsa-miR-135a-5p			-2.65	0.0076		
hsa-miR-135b-5p	-5.61	0.0002	-4.86	0.0007		
hsa-miR-146b-5p					3.29	0.0322
hsa-miR-148b-3p	2.92	0.0144	3.65	0.0029		
hsa-miR-151a-5p	-4.89	0.0041				
hsa-miR-152-3p			7.62	<0.0001	-2.61	0.0130
hsa-miR-15b-5p	-3.45	0.0076	-2.70	0.0273		
hsa-miR-181a-3p	5.57	0.0012	5.36	0.0010		
hsa-miR-181a-5p			-1.96	0.0487		
hsa-miR-185-5p					-2.10	0.0199
hsa-miR-191-5p	-2.92	0.0009	-2.56	0.0025		
hsa-miR-195-5p					-2.70	0.0281
hsa-miR-19a-3p			6.80	0.0034	-2.12	0.0482
hsa-miR-19b-3p			3.75	0.0100	-1.06	0.0495
hsa-miR-200c-3p			2.69	0.0218		
hsa-miR-204-5p					-2.87	0.0465
hsa-miR-221-3p					3.80	0.0343
hsa-miR-222-3p					2.46	0.0192
hsa-miR-20a-5p + hsa-miR-20b-5p	-3.08	0.0222	-3.29	0.0030		
hsa-miR-25-3p			2.12	0.0487		
hsa-miR-28-5p	-2.61	0.0123			-1.72	0.0394
hsa-miR-296-5p					-2.40	0.0130
hsa-miR-301a-3p	4.18	0.0136	5.55	0.0273		
hsa-miR-30c-5p	-6.18	0.0003	-5.22	0.0004		
hsa-miR-30d-5p	-2.92	0.0123	-2.78	0.0025		
hsa-miR-30e-3p			5.61	0.0281	-2.97	0.0130
hsa-miR-30e-5p	-3.90	0.0037	-3.00	0.0042	-0.90	0.0495
hsa-miR-3151-5p			-3.63	0.0025		
hsa-miR-32-5p	-6.61	0.0002	-5.93	0.0011		
hsa-miR-331-3p					-2.30	0.0322
hsa-miR-340-5p					-2.25	0.0458
hsa-miR-34a-5p	-4.40	0.0006	-4.55	<0.0001		
hsa-miR-361-3p					-2.59	0.0192
hsa-miR-361-5p	-5.47	0.0076	-5.38	0.0001		
hsa-miR-363-3p					-2.20	0.0488
hsa-miR-365a-3p + hsa-miR-365b-3p					-2.51	0.0343
hsa-miR-374b-5p	-4.54	0.0158			-1.80	0.0495
hsa-miR-423-3p	-5.21	0.0002			-2.55	0.0130
hsa-miR-424-5p			-4.66	0.0025		
hsa-miR-4286	4.02	0.0173	3.89	0.0218		
hsa-miR-429	8.65	0.0006	8.46	0.0010		
	log FC	FDR	log FC	FDR	log FC	FDR
hsa-miR-4454 + hsa-miR-7975					1.28	0.0343
hsa-miR-4516					2.33	0.0480
hsa-miR-451a			2.37	0.0218		
hsa-miR-454-3p			5.50	0.0174	-2.36	0.0281
hsa-miR-518b					-3.24	0.0130
hsa-miR-5196-3p + hsa-miR-6732-3p					-2.83	0.0192
hsa-miR-660-5p					-2.30	0.0385
hsa-miR-98-5p	-2.20	0.0135	-1.88	0.0218		

Abbreviations: FAs, follicular adenomas; FVPTCs, follicular variant of papillary thyroid carcinomas; FC, fold change; FDR, false discovery rate.

### mRNA expression levels

**Figure 6:**
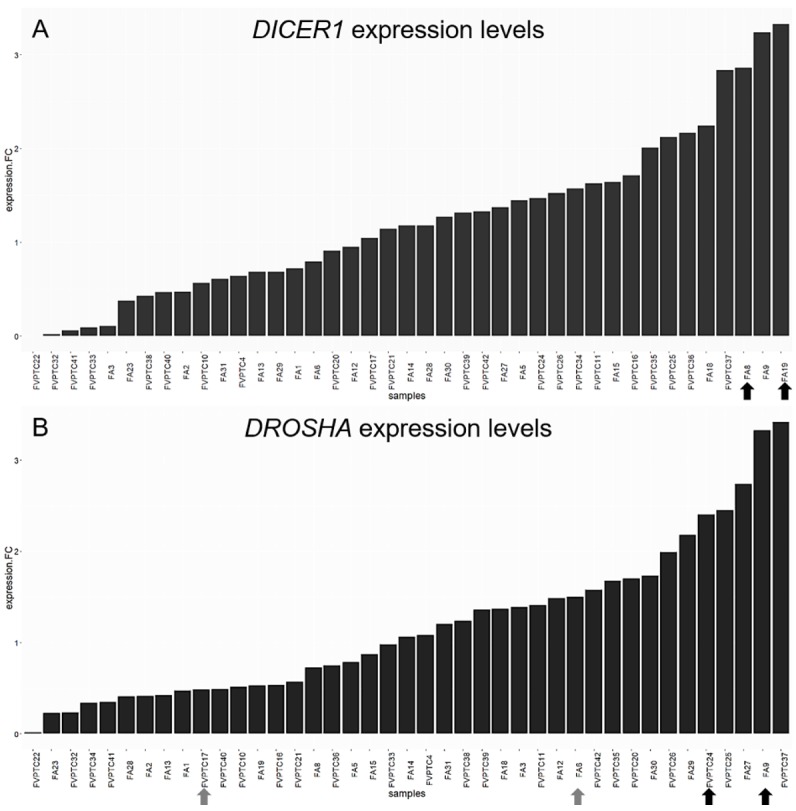
*DICER1* and *DROSHA* expression levels Histograms represent the log-fold change expression of *DICER1* **(A)** and *DROSHA* **(B)** mRNA calculated by the ΔΔC_t_ method. The two *DICER1* mutants (A) and *DROSHA* p.Y1199Y (B) carriers are emphasized by black arrows. Grey arrows highlight *DROSHA* p.S981S carriers.

The expression levels of *DGCR8*, *DICER1*, *DROSHA*, *TARBP2* and *XPO5* were not significantly different between FAs and FVPTCs. However, the two *DICER1* mutated FAs had high mRNA levels of *DICER1* (Figure 6A). Likewise, the two lesions harboring *DROSHA* p.Y1199Y variant had high levels of *DROSHA* mRNA (Figure 6B).

### TCGA data analysis

Three TCGA cases harbored *DICER1* mutations (0.6%); two of these cases were in the RNase IIIb domain (one p.E1813G and one p.D1810H) and one in the double strand RNA-binding domain (p.R1906S). The mutations in the RNase IIIb domain did not coexist with *RAS* or *BRAF* mutations, whereas the case harboring *DICER1* p.R1906S had a concomitant *NRAS* p.Q61R. Further details are reported in Table 3. Two cases (0.5%) harbored mutations in the double strand RNA-binding domain of *DGCR8* (two p.E518K), but they both had a concomitant *NRAS* mutation. One of the two cases harboring *DGCR8* p.E518Kshowed no consistent loss of 5p miRNAs.

**Table 3:** TCGA data analysis of *DICER1* mutant cases

ID	TCGA-EL-A3D5		TCGA-EL-A3GO		TCGA-EM-A2CT	
Histology	CVPTC		CVPTC		FVPTC	
*DICER1* mutation	E1813G		D1810H		R1906S	
*DICER1* mutation domain	RNase IIIb		RNase IIIb		double-stranded RNA binding	
Other mutations	*TP53* *NCAN* *NAB2* *ABCB11* *RPAP1* *TSPYL2* *AMDHD1*	E343Afs^*^3 P1274S L495Cfs^*^22 Q216^*^ L628R X413_splice D334Ifs^*^20	*SHQ1* *ARHGEF6* *SLITRK3* *TECPR1* *DNAH1* *HEATR6* *DYNC2H1* *SBF2* *CSMD2* *CCDC24* *OR51F1* *STAC2*	D13H W439C R809W W763Gfs^*^56 R4133S K437N G1396Efs^*^2 I1545S M2857T A44V K306N E241K	*NRAS* *IRS1* *TP73* *SYNJ2* *OSMR* *WDR7* *NGEF* *KRTAP4-12* *SLC9A4*	Q61R M664I E40G S1032Y D692G X1277_splice L681P R192H K622N
Copy number alteration	*PDE3A*	AMP	*PTPRT*	AMP	*DUSP22* *IRF4* *EXOC2* *HUS1B* *SLC8A1* *CBLN2* *NETO1* *LINC01541* *LOC400655* *LINC02582*	AMP AMP AMP AMP DEL AMP AMP AMP AMP AMP
Putative driver	*DICER1* E1813G		*DICER1* D1810H		NRAS Q61R	
*DICER1* mRNA expression percentile	99.18		99.59		60.29	
5p miRNA percentage percentile	0.18		0.53		7.03	

Abbreviations: CVPTC, classic variant of papillary thyroid carcinoma; FVPTC, follicular variant of papillary thyroid carcinoma; AMP, amplification; DEL, deletion.

## DISCUSSION

In the deep sequencing era, there is an urgent need for the discovered mutations to be contextualized so that they may be helpful in the clinical practice. Thyroid cancer is a paradigm for the use of molecular information in the clinical field, especially for diagnostic purposes. Follicular-patterned lesions are the major challenge in the pre-surgical diagnosis of thyroid nodules; for this reason, they are frequently sequenced in order to find mutations that could help to distinguish benign from malignant lesions [8–10]. This deep sequencing unearthed several somatic mutations previously unknown. Among these, *DICER1* mutations were formerly known as germline mutations, which are associated with the so-called *DICER1*-syndrome and predispose carriers not only to multinodular goiter but also to pleuropulmonary blastoma, cystic nefroma and embryonal rhabdomyosarcoma. Although somatic *DICER1* mutations have not been extensively studied in thyroid cancer, it is accepted that they are driver events [6, 7]. However, their potential usefulness in the clinical practice is still questionable, also because they are shared by both benign and malignant lesions [9]. In this scenario, we sought to contextualize genetic alterations in *DICER1* and in the other miRNA processing genes concerning the molecular dysregulations they produce, in order to provide important information for clinical decisions in presence of lesions harboring these alterations.

Our series confirmed that somatic *DICER1* mutations have never coexisted with the most common driver mutations in thyroid cancer. However, the two *DICER1* mutated cases were FAs: despite the benign nature of these lesions, PCA highlighted the entity of miRNA dysregulation (Figure 1), and the miRNA profile of the two lesions harboring *DICER1* p.D1810V and p.E1813K was very similar (Figure 2). The dysregulation mainly affected 5p miRNAs (Figure 3), as previously reported in other cancer models [11]. In spite of the low number of *DICER1*-mutants and the consequent low statistical power, several miRNAs were deregulated when contrasting FAs harboring *DICER1* mutations with both FVPTCs and FAs (Table 2). Consistently with the previous analysis, all differently expressed 5p miRNAs were downregulated in *DICER1*-mutant group with respect to FAs as well as FVPTCs. Interestingly, there was very little overlap between miRNAs deregulated in FVPTCs and in *DICER1*-mutants versus FAs (Figure 4). Moreover, 15 miRNAs were specifically deregulated in *DICER1*-mutants. These miRNAs were then tested for pathway enrichment analysis to identify pathways affected by such a deregulation. The analysis showed that these miRNAs are crucial in several pathways (Figure 5), and not only metabolic ones or pathway fitting benign conditions such as fatty acids metabolism, but they also regulate pathways with a pivotal role in cancer, such as Hippo, p53 and TGF-beta [16, 17]. In addition, adherens junction and proteoglycan pathways also seem to be affected, and they are known to be involved in the malignant transformation due to the gaining of an invasive potential [18, 19].

As concerns *DROSHA* germline mutations, p.S981S and p.Y1199Y have been previously described in both ExAC and 1000 Genomes, in which individuals with evident diseases have been removed. However, the frequencies of these variants were considerably lower than the 0.01 cut-off value for polymorphisms, but were much higher in our series. This difference could be due to chance, but there was also an interesting trend for these *DROSHA* germline mutant carriers to be younger than the other patients were.

Indeed, our study suffers from some limitations such as low number of samples, especially *DICER1* mutants. Although we did not perform a functional analysis of *DICER1* mutations, our results are consistent with the findings of Anglesio *et al.* regarding engineered mice embryonal stem cells, in which mutations affecting the heme-binding residues in the RNase IIIb domain of DICER1 resulted in a loss of mature 5p miRNAs [11]. Moreover, we confirmed our findings with TCGA data, in which cases harboring *DICER1* mutations in the RNase IIIb domain never coexisted with other well-known driver mutations in thyroid cancer. Although these cases had a high expression level of *DICER1* mRNA, they had a remarkable reduction in mature 5p miRNAs (Table 3).

**Figure 7:**
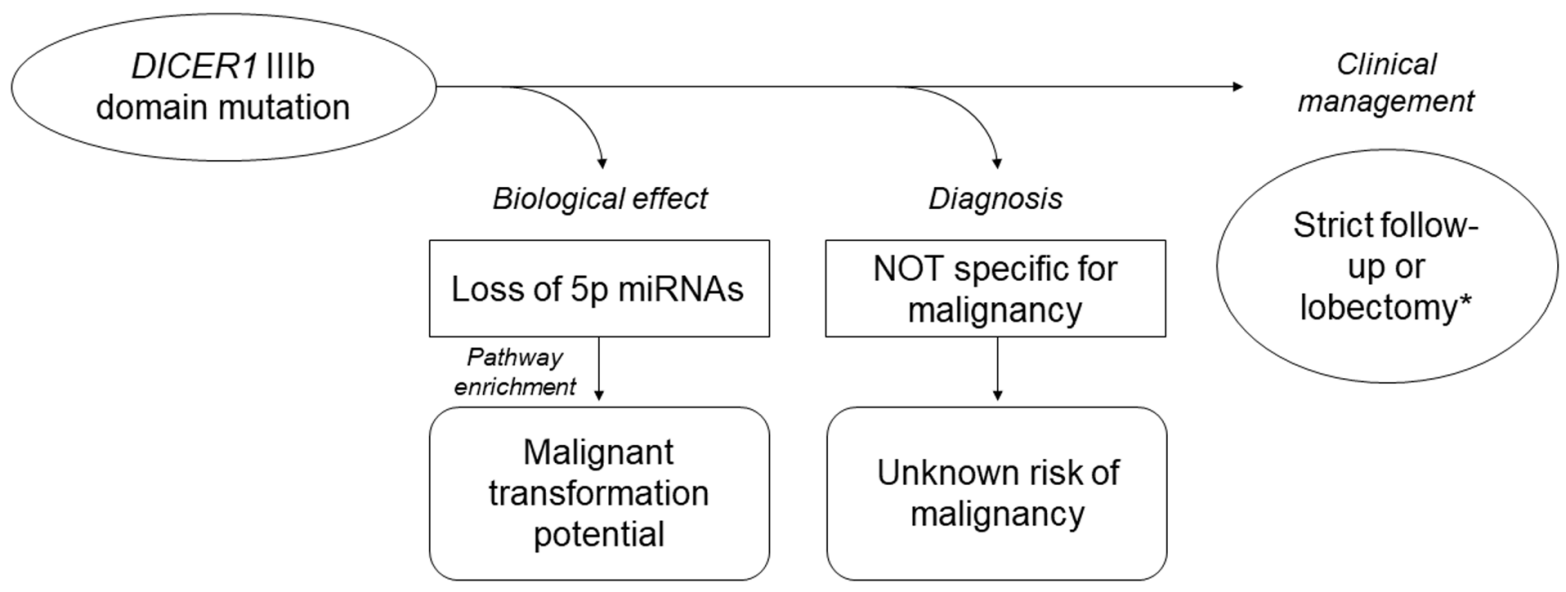
Current knowledge of *DICER1* IIIb somatic mutations in thyroid tumors based on the present study results and on the literature data Owing to the lack of data, the impact of these mutations on prognosis is unknown. ^*^based on the clinical context.

To sum up, our study provided further evidences of the involvement of the miRNA processing genes and of miRNA dysregulation in thyroid cancer. There are some points that need to be highlighted: a) *DROSHA* rs17485810 and rs61748189 germline variants could be predisposing, but not sufficient factors for thyroid tumors, as demonstrated by the co-occurrence of *RAS* mutations; b) *DICER1* somatic mutations are not specific markers for malignancy, and further studies providing sufficient follow-up data, real frequencies and, most importantly, the risk of malignancy associated with them are needed; c) *DICER1* somatic mutations in the RNase IIIb domain strongly impair miRNA processing; d) the deregulation of miRNAs in *DICER1*-mutants is peculiar and different from that in FVPTCs. When detected preoperatively, *DICER1* mutations could then suggest a surgical approach to these lesions, since tumors harboring *DICER1* mutations are very different from wild-type ones, regardless of their benign or malignant nature, and similarly to *RAS* mutants they conceal deep molecular alterations with the potential of evolving in malignant forms (Figure 7).

In conclusion, although sufficient follow-up data of *DICER1*-mutant lesions is necessary to provide a definitive answer to this issue, the remarkable molecular alterations caused by these mutations should be taken into account in clinical decision-making.

## MATERIALS AND METHODS

### Study population

The study included 41 samples retrospectively collected from the archives of the section of Pathology of the University Hospital of Pisa. Only the most com-mon follicular-patterned lesions were selected—i.e., FA and FVPTC. Hematoxylin and eosin stained sections were independently reviewed by two pathologists (F.B. and L.T.). The lesions were diagnosed according to the World Health Organization 2017 diagnostic criteria [20]. FAs were submitted *in toto* to exclude the presence of vascular or capsular invasion. The study conformed to the principles of the Helsinki Declaration of 1975, it was conducted anonymously, and no sensitive data was used.

### Nucleic acids purification and mutational analyses

For each sample, four unstained 10μm- and 5μm-thick formalin-fixed and paraffin-embedded (FFPE) sections were used for DNA and RNA purification, respectively. Unstained sections were deparaffinized in xylene and then rehydrated in ethanol solutions. After manual macro-dissection, DNA and total RNA were isolated by the QIAamp DNA Mini Kit (Qiagen Inc, Hilden, Germany) and the miRNeasy FFPE Kit (Qiagen Inc, Hilden, Germany) respectively, according to the manufacturer’s protocols. Quantity and quality of nucleic acids were assessed by spectrophotometry (Xpose Trinean, Gentbrugge, Belgium).

The mutational status of *BRAF* (exon 15), the *RAS* genes (exons 2–3), *DICER1* (exons 24–25), *DROSHA* (exons 23–24,29–30), *DGCR8* (exon 7) and *TARBP2* (exons 7–8) were analysed by direct sequencing (3130 Genetic Analyzer, Applied Biosystems, Foster City, CA, USA), according to standard procedures [21]. Primers used for sequencing miRNA processing genes are reported in Table 4. In addition, the presence of *PAX8-PPARG* rearrangements (exons 7 and 9) was investigated, as previously described [22]. The paired normal tissues from the contralateral lobe or the normal collateral parenchyma of lesions harboring mutations in the miRNA processing genes were used to confirm the germline or somatic nature of the variants according to current guidelines [23]. To ensure that the tissue was normal, without tumor foci, sections above and below those used for nucleic acids isolation were stained with hematoxylin and eosin and independently reviewed by two pathologists.

**Table 4:** Primers used for sequencing selected exons of the miRNA processing genes

Gene	Transcript ID	Exon	Functional domain	Primers
*DICER1*	ENST00000343455.7	24	RNase IIIb	F: AAGCTTACGGTTCCACTTCG
				R: ACCACTATGCCGTCAGAACT
		25		F1: AGAATAATAAATGGGGTGGGGAT
				R1: GTACACCTGCCAGACTGTCT
				F2: AACTACATCTGTGGACTGCC
				R2: TCAAGCAATTCTCGCACAGG
*DROSHA*	ENST00000511367.6	23	RNase IIIa	F: TGTGTTCTTTTCTTGCGGGG
				R: ACACGGTGTATCAATGCCTT
		24		F: TCAGAGCCACAGACAGAATGT
				R: ACCGCAGAAGAGCATGTCA
		29	RNase IIIb	F: TCAATCGAGGGGCCTTAGG
				R: TGGAGGAAGTGATTTAACCAACT
		30		F: TGTTCTCAATGTACCGCCAT
				R: AGGAGGAGGACAAATACGGT
*DGCR8*	ENST00000351989.7	7	heme- and pri-miRNA binding	F: GTGGCACTGCTTCACACTTG
				R: GCCCTGACCAAAGTTACACC
*TARBP2*	ENST00000266987.6	7	DICER1- and pre-miRNA binding	F: GGTCTGTGGGGAATCATAACC
				R: CAGAAGCAGACCTAGGGCC
		8		F: TCGCTTCATCTTTCTCACTGT
				R: CCCTTCTACTTGCTCCGGTC

### Expression analyses

The expression profiling of 798 miRNAs was carried out by the nCounter human v3 miRNA expression assay (NanoString Technologies, Seattle, WA, USA), as previously described [24].

The expression levels of *DICER1*, *DROSHA*, *TARBP2*, *DGCR8* and *XPO5* were evaluated by RT-PCR using the QuantiTect Primer Assay (Qiagen Inc, Hilden, Germany), and following the manufacturer’s instructions. In detail, the retrotranscription step was performed using the RevertAid First Strand cDNA Synthesis Kit (Thermo Scientific, Waltham, MA), as previously reported [22]. Fifty ng of cDNA were then used in a reaction volume of 25 μL with the Rotor-Gene SYBR Green PCR Master Mix (Qiagen Inc, Hilden, Germany), and with specific primers. Amplification was performed in 40 cycles (denaturation at 95°C for 5 sec, annealing and elongation at 60°C for 10 sec). Actin beta (*ACTB*) and glyceraldehyde-3-phosphate dehydrogenase (*GAPDH*) were used as housekeeping genes, and thyroglobulin (*TG*) was used as specificity control. Each sample was amplified in double copy. The ΔΔC_t_ method was used to assess the relative quantification of the targets [25].

### Statistical analyses

miRNA expression counts were normalized according to the manufacturer’s instructions. In detail, background thresholding was applied to raw counts by using the max value of the negative control counts as threshold. In addition, only miRNAs with counts above the threshold in more than 60% of samples were considered for further analyses. PCA was used to summarize the variation pattern of the dataset. Hierarchical clustering was performed using Pearson’s correlation and average as distance and clustering functions, respectively. The moderated t-statistics was used to evaluate the miRNA differential expression. After normal distribution assessment by the Shapiro-Wilk’s test, continuous variables were tested by the two-tailed Student’s *t* test. Fisher’s exact test was used to analyse categorical variables. miRNA pathway analysis was performed by the mirPath v.3 web server [26]; for the analysis, KEGG and TarBase v.7.0 were used as pathway and miRNA reference databases, respectively. In addition, the analysis was performed using the *a posteriori* method “pathway union” by a modified Fisher’s exact test. All p values were adjusted with the Benjamini-Hochberg’s correction, and FDR of 0.05 was set as significance level. The prediction of the effect of uncommon mutations was conducted by Mutation Taster [27]. The mutational status of *DGCR8*, *DICER1*, *DROSHA* and *TARBP2* of thyroid cancer TCGA cases was explored by using the cBioportal (http://www.cbioportal.org); level 3 miRNA expression data was downloaded from FireHose (http://gdac.broadinstitute.org). All statistical analyses were performed in R environment (http://www.R-project.org).

## Abbreviations

ExAC: Exome Aggregation Consortium
FA: follicular adenoma
FDR: false discovery rate
FFPE: formalin-fixed and paraffin-embedded
FVPTC: follicular variant of papillary thyroid carcinoma
KEGG: Kyoto Encyclopedia of Genes and Genomes
PCA: principal component analysis
TCGA: The Cancer Genome Atlas
FA: follicular adenoma
FDR: false discovery rate
FFPE: formalin-fixed and paraffin-embedded
FVPTC: follicular variant of papillary thyroid carcinoma
KEGG: Kyoto Encyclopedia of Genes and Genomes
PCA: principal component analysis
TCGA: The Cancer Genome Atlas
